# Not Getting Vaccinated? It Is a Matter of Problem-Solving Abilities and Socio-Cognitive Polarization

**DOI:** 10.3390/ijerph20031721

**Published:** 2023-01-17

**Authors:** Alice Cancer, Carola Salvi, Alessandro Antonietti, Paola Iannello

**Affiliations:** 1 Department of Psychology, Università Cattolica del Sacro Cuore, 20123 Milan, Italy; 2 Department of Psychiatry and Behavioral Sciences, University of Texas at Austin, Austin, TX 78712, USA; 3 Department of Psychology and Social Sciences, John Cabot University, 00165 Rome, Italy

**Keywords:** anti-COVID-19 vaccine, problem solving, cognitive flexibility, socio-cognitive polarization, political beliefs

## Abstract

The anti-COVID-19 vaccination campaign in the United States provided a significant contribution to the control of the virus spread. Despite the recommendations by public health institutions, vaccine skepticism and hesitancy contributed to low vaccine uptake, thus possibly disrupting the management of preventable diseases associated with the COVID-19 infection. The process that led individuals to accept COVID-19 vaccines required the ability to gather, synthesize, and weigh-up information within a novel, dynamically changing, complex, and ambiguous context. To deal with such complexity, we hypothesized that both the ability of reflection and flexible adaptation played a fundamental role. Based on previous research on cognitive predictors of vaccine refusal, we decided to investigate the combined role of two constructs, namely, problem-solving skills and socio-cognitive polarization (SCP), on vaccine acceptance and uptake. Two-hundred-seventy-seven US participants completed an online survey aimed to measure problem-solving ability, through a rebus puzzles task, and SCP, through a composite measure of absolutist thinking, political conservatism, and xenophobia. Mediation analyses indicated that SCP mediated the association between problem-solving ability and vaccine acceptance, so lower problem-solving abilities associated with higher polarization predicted vaccine rejection. Thus, our findings suggested that low problem-solving skills may represent a risk factor for COVID-19 vaccine refusal, with cognitive and social rigidity playing a crucial role in undermining the anti-COVID-19 vaccine uptake.

## 1. Introduction

The COVID-19 pandemic caused over 1.08 million deaths in the United States over the course of the past two years [1]. A mass vaccination campaign began in December 2020, immediately after the Pfizer–BioNTech vaccine was granted emergency use authorization by the Food and Drug Administration (FDA) [2]. After an initial period of selective vaccination for the elderly and other vulnerable populations, the vaccine was made available to all adults, and, by July 2021, 67% of the United States’ adult population had received at least one dose [3]. Before September 2021, no federal vaccine mandate was enacted; Nonetheless, vaccination mandates have largely been enforced by private businesses and event promoters, who required proof of vaccination from employers and customers [4].

During emergencies, conditions rapidly change, requiring constant adaptation to new situations and immediate problem solving. The COVID-19 pandemic was a novel, unexpected, and demanding situation that called for new solutions at the individual and societal levels [5,6]. The ability to develop novel strategies to solve emerging problems relies in large part on cognitive flexibility. Flexible adaptation of behavior in the face of changing environments is a core feature of human cognition, especially when confronted with a multifaceted and rapidly changing world.

Problem solving is an established measure of cognitive flexibility that translates into the ability to generate novel and original ideas by exploring unusual reasoning paths and challenging rigid perspectives around us [7,8]. Previous studies showed that problem solving is associated with flexible political perspectives, tolerance of diversity, and fake news discernment [9,10,11,12]. Such evidence supports the idea that problem solving is related to a broader capacity for social reasoning and environmental information processing [10,13], thus promoting adaptive behaviors in complex and critical situations.

Recently, we introduced the construct of *socio-cognitive polarization* (SCP), a factor capturing conservative political ideology, absolutist thinking, intolerance of ambiguity, and xenophobia (for more details see [10]). The shared literature on emotional, social, and cognitive factors underlying political conservatism, intolerance for ambiguity, and xenophobic reactions suggests that people who score higher on these measures may be less likely to handle complexity and thus fail to seek out alternative explanations when processing information [10]. When facing complex situations, rigid individuals may be more prone to use heuristics and mental shortcuts [14]—which ultimately are an (over)simplification of reality—to arrive at a quick solution. Consistently, our previous results showed that the SCP construct is related to reasoning processes based on thinking outside the box and considering alternative information such as problem solving [10].

Based on this association, it can be hypothesized that, in the context of the COVID-19 pandemic, individual differences in problem-solving abilities and socio-cognitive rigidity would reflect differences in vaccination hesitancy.

The World Health Organization’s Strategic Advisory Group of Experts (SAGE) defined vaccine hesitancy as the “delay in acceptance or refusal of vaccination despite the availability of vaccination services’’ [15]. Considering the anti-COVID-19 vaccine, early explorations of vaccine skepticism evidenced a correlation with female gender, low income, social media misinformation or exaggeration and conspiracy theories, government mistrust, and anti-government sentiment [16], as reported in studies conducted in various countries, e.g., Australia [17], France [18], Greece, [19], Israel [20], Italy [5,21], USA [22]. Further, vaccination uptake is significantly related to people’s political orientation. Research indicated that more conservative, right-leaning political views are more likely to report stronger anti-vaccination attitudes and lower vaccination uptake [23,24,25]. Finally, cognitive rigidity, measured as absolutist thinking, was confirmed as a predictor of parents’ hesitancy to vaccinate their young children [5].

To our knowledge, problem-solving skills were never studied in relation to vaccine hesitancy in the context of COVID-19. Thus, in this study we aimed to investigate the combined role of problem solving and socio-cognitive rigidity on vaccine acceptance and uptake. More precisely, we hypothesized that cognitive rigidity would mediate the association between problem solving and vaccine acceptance, so that lower problem-solving abilities associated with higher rigidity would predict an anti-vaccine stance.

## 2. Materials and Methods

### 2.1. Procedure

Data were collected from February to June 2021 in the US. The link to an online survey hosted on the Qualtrics platform was shared via Amazon’s Mechanical Turk (MTurk) platform using CloudResearch [26]. To ensure data quality, only pre-vetted workers who had demonstrated attention and engagement were allowed to participate. The study was restricted to individuals between the ages of 18 and 50, who were located in the United States and spoke American English as their first language. Only people who passed this initial selection were allowed to participate in the study. The selection was made via MTurk’s hits and qualification requirements. The study took approximately one hour to complete (with a median completion time of 42.3 min) and participants received a total of USD 4 upon completion. The study was approved by the IRB at the University of Texas at Austin and all participants provided written informed consent.

Participants were first directed to an informational page and online consent form. Following written consent, participants completed a short demographic survey, the experimental paradigm, and a series of questionnaires. Each of these components is detailed below.

### 2.2. Measures

#### 2.2.1. Demographics

Respondents were asked to share their socio-demographic information (i.e., age, sex, marital status, and educational level).

#### 2.2.2. Vaccine Acceptance

Vaccine acceptance was measured using 2 items investigating: (1) COVID-19 vaccination status (‘Are you vaccinated against COVID-19?’) and (2) attitude towards the COVID-19 vaccine (‘Are you in favor of the COVID-19 vaccine?’). To be considered for the present investigation, participants must have demonstrated a clearly defined and coherent approach toward COVID-19 vaccines. More precisely, only participants who provided matching responses to the behavioral (a) and attitudinal (b) aspects of vaccine acceptance were included in the study.

#### 2.2.3. Socio-Cognitive Polarization (SCP)

SCP, as described by Salvi and colleagues [10], was measured by averaging scores obtained in conservatism [27], absolutism [28], and xenophobia [29]. More precisely, a measure of conservatism was calculated as a function of conservative political ideology endorsement (“I endorse many aspects of conservative political ideology”) minus liberal political ideology endorsement (“I endorse many aspects of liberal political ideology”) [9,12,27]. Absolutism, conceptualized as rigid and dichotomous thinking, was measured through the Moral Absolutism/Splitting subscale from the Multidimensional Attitude Toward Ambiguity Scale (MAAS; “There are two kinds of people: the ‘good’ and the ‘bad’”) [28]. Xenophobia, representing hostility and fear toward immigrants, was measured using van der Veer and colleagues’ [29] Xenophobia Scale (e.g., “Interacting with immigrants makes me uneasy”).

#### 2.2.4. Creative Problem Solving

Creative problem solving was measured through the online version of rebus puzzles [30]. Several studies, both online and in person, showed that these types of puzzles are an established measure of insight problem solving (e.g., [10,31,32]). We decided to study creative problem solving specifically because the respondent is required to consider multiple viewpoints and to deal with multiple options to reach the correct solution. Similar creative abilities are involved in the vaccine decision-making process within a complex and constantly changing context such as the COVID-19 pandemic. The task, originally developed by MacGregor and Cunningham [33], requires participants to identify a common phrase from the verbal and visual clues provided on screen (e.g., ‘cycle, cycle, cycle’ would be solved as ‘tricycle’). Forty-four randomized trials (one rebus per trial) were presented to the participants. The time limit for each trial was set at 15 s. Two types of errors were recorded, (a) omissions (i.e., no solution was given) and (b) commissions (i.e., the incorrect solution was given). Following the procedure applied by Cancer et al.’s [34], a rebus performance criterion (C) index was calculated by subtracting the number of commission errors from that of correct responses and dividing it by the number of total rebus problems. If C > 0, a higher number of correct responses was generated, whereas if C < 0, a higher number of incorrect responses was generated.

### 2.3. Participants

Three hundred and forty-four participants completed all sections of the survey. Of these, 277 were selected based on the coherence of their vaccine acceptance responses (i.e., vaccination status and attitude toward the vaccine). This selection was made to exclude participants who did not show a clear stance on COVID-19 vaccination. Thus, only participants who could be assigned to either the pro-vax (i.e., vaccinated and in favor of the vaccine) or the anti-vax group (i.e., unvaccinated and against vaccination) were included in the study. The socio-demographic characteristics of the final sample are reported in Table 1.

### 2.4. Statistical Plan

First, descriptive statistics of participants’ characteristics were calculated (Table 1).

Second, a mediation analysis was performed using PROCESS (Model 4; [35]) to assess the mediating role of SCP on the association between creative problem solving and vaccine acceptance.

## 3. Results

**Figure 1:**
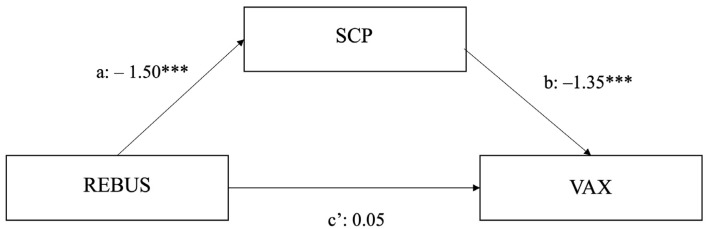
Mediating effect of SCP on the relationship between creative problem solving and vaccine acceptance (VAX). *** *p* < 0.001. [a = direct effect of REBUS on SCP; b = direct effect of SCP on VAX; c’ = direct effect of REBUS on VAX after controlling for SCP].

The mediation analysis revealed a significant indirect effect of creative problem solving (REBUS) on vaccine acceptance (VAX) through SCP (*b* = 2.04, SE = 0.44; 95% CI [1.30, 3.05]). The model revealed a nonsignificant effect of problem solving on VAX after controlling for SCP (*b* = 0.05, SE = 0.68; 95% CI [–1.28, 1.39]), thus implying full mediation (Figure 1). This result revealed that participants’ performance in the creative problem solving task did not have a direct significant impact on vaccine acceptance, but had a significant impact on SCP, which also had a significant impact on vaccine acceptance.

## 4. Discussion

The anti-COVID-19 vaccine provided a significant contribution to the COVID-19 pandemic [36]. However, the existence of vaccines does not automatically lead to their acceptance [37]. The process that leads individuals toward the acceptance of vaccines can be generally regarded as a quite complex set of assignments that require the ability to acquire, synthesize, and weigh-up information [38]. During the COVID-19 pandemic, deciding on vaccination was made even more difficult by the fact that the situation exhibits the specific characteristics of being novel, dynamically changing, complex, and ambiguous. Dealing with such a context implied the capacity for reflection and flexible adaptation.

In our study, the prevalence of pro-vax individuals among participants was 71.1%, whereas that of anti-vax individuals was 28.9%. Such distribution reflected the percentage of the US population above 18 years of age who completed the primary series of the anti-COVID-19 vaccine (≈79%) [3]. US anti-COVID policies differed from those adopted by European governments, who introduced vaccination mandates for selected populations (e.g., health-care workers, teachers, seniors over 60 years) and required a COVID pass demonstrating immunization status (i.e., recent infection or vaccination) for working, travelling, and indoor activities and public services [39]. The absence of a governmental mandate in the US, likely due to the decentralized system of government and a long history of anti-vaccine sentiments, has to be taken in account when investigating anti-COVID vaccine hesitancy.

The main aim of the present contribution was to investigate the combined role of problem solving and socio-cognitive polarization in vaccine acceptance, specifically in the context of the United States.

Problem-solving skills can be an important factor in addressing inflexible attitudes and promoting more open-minded approaches to evaluating new information, including information about vaccines. The ability to consider multiple viewpoints and to adapt to new information can be important for making informed decisions and for avoiding the pitfalls of cognitive biases and dogmatic thinking [40]. Research on the relationship between problem-solving skills and attitudes toward vaccination is still scarce. One study by Senger and Huynh [41] found that individuals with higher levels of intellectual humility, namely, the nonjudgmental recognition of one’s intellectual fallibility, were less likely to hold negative attitudes towards vaccines. However, it is important to note that the construct of intellectual humility does not necessarily overlap with problem-solving skills, thus more research is needed to further understand the relationship between problem-solving skills and attitudes toward vaccines.

In our study, we included a measure of socio-cognitive polarization that goes beyond the “pure” and well-known cognitive dimension of rigidity [10]. From previous research [10,11] we know that cognitive rigidity is often expressed in forms of social rigidity, where people who score high on problem solving (cognitive flexibility) also show flexible political perspectives, tolerance of diversity, and fake news discernment. This association suggests that the construct of rigidity extends beyond its mere cognitive manifestation to also include a social dimension. Consistently, previous evidence showed that individuals with more conservative political ideologies tend to be more cognitively rigid and less open to new information and alternative viewpoints. Other research has found that individuals who are more politically partisan tend to be more resistant to changing their beliefs, even in the face of new evidence [9,10,11,42,43]. Based on such evidence and consistently with our novel results, social and cultural factors, including political ideology and beliefs, play a role in attitudes towards vaccination. As reported in the literature, anti-vaccine attitude is linked to a specific political orientation and social/racial beliefs, with more conservative, right-leaning people being more likely to develop anti-vaccination attitudes and lower vaccination uptake [23,24]. This tendency can be explained by the widespread belief in a limited role of the US government in managing the affairs of citizens [44]. Although anti-vax positions are predominantly right-leaning, also left-oriented anti-vax movements exist, yet they appear to be less substantial [45]. Pre-pandemic research suggests that in the US “the more ideologically extreme a respondent is (regardless of whether conservative or liberal) the more likely the person is to think that the vaccines are unsafe” [44]. However, in the context of the COVID-19 pandemic, our results showed conservative beliefs, clustered in the larger construct of SCP, to be specifically associated with anti-vaccine attitudes.

Furthermore, the findings of our study indicate that problem solving did not have a direct effect on vaccine acceptance, after controlling for SCP; Rigidity mediates the association between problem-solving and vaccine acceptance so that lower problem-solving abilities associated with higher rigidity would predict vaccine rejection. Problem-solving skills do not uniquely explain vaccine acceptance; People with low problem-solving abilities probably perceive that they are not equipped to effectively face a complex and ambiguous situation such as an unprecedented global pandemic. When facing this complex situation, individuals with low problem-solving abilities and high levels of polarization tend to adopt strategies aimed at simplifying reality. When poor problem-solving skills are associated with rigidity (high polarization), this probably implies the tendency to rely on absolutist and dichotomous thinking to make reality more understandable and manageable, even though this may lead to an oversimplification of the situation. The combined effect of scarce problem-solving abilities and high rigidity makes people more likely to refuse vaccines. In a novel, dynamically changing, complex, and ambiguous context, we suggest that polarization was used by poor problem-solvers to make the vaccination decision more understandable, more manageable, and simpler, and ultimately directed them toward a negative attitude toward vaccination. The cognitive facet of rigidity was indeed previously shown to hinder COVID-19 vaccine acceptance and mistrust of its uptake [5].

In short, our results suggest that low problem-solving skills are a factor that mediates COVID-19 vaccine refusal, with cognitive and social rigidity playing a fundamental role to undermine the vaccine uptake.

Finally, it is worth mentioning that there are a few limitations to the present study. First, using a web-based survey will not reach people who are not familiar with the technology. Second, due to the cross-sectional design of our study, the potential effect of the updated reports about COVID-19 vaccine safety and efficacy on vaccine acceptance rates was not investigated.

## 5. Conclusions

Anti-vaccine attitudes can indeed have serious consequences, particularly during a global pandemic such as the current COVID-19 one [46,47]. Vaccines are one of the most effective tools we have for controlling infectious diseases and protecting public health and low vaccine uptake can lead to outbreaks of preventable diseases.

There has been a significant increase in social media activity related to anti-vaccine sentiment and COVID-19 and this can contribute to the spread of misinformation and mistrust in vaccines (e.g., [48,49]). It is important for researchers to identify psychological factors that may generate inflexible attitudes toward vaccines and to develop strategies for combating these attitudes and promoting vaccine uptake. This may involve addressing concerns and providing accurate information about vaccines, as well as addressing social and cultural factors that may influence attitudes toward vaccination.

Our results suggest that individual characteristics such as low problem-solving skills combined with high rigidity on both cognitive and social levels may have hindered vaccine acceptance in the context of the COVID-19 pandemic. More precisely, individuals have to rely on their problem-solving skills to weigh up and update the constantly changing body of information related to the effects and safety of the COVID-19 vaccine to ponder their decision on the uptake. In addition to that, absolutist thinking and social rigidity appear to hinder the flexible adherence to new vaccine recommendations through the polarization towards a status quo defending anti-vaccine attitudes, despite scientific evidence and public health institutes’ directions.

In conclusion, we suggest that it is fundamental to consider specific psychological determinants of vaccine hesitancy. Identifying the psychological dimensions behind vaccine refusal may offer evidence-based strategies to promote more effective health communication campaigns to support, promote, and strengthen vaccine uptake. More broadly, our results might be useful to plan and ground strategies in health education that are deeply rooted in evidence-based data.

## Figures and Tables

**Table 1:** Participants’ characteristics.

	*n* (%)
**Age** ^1^	34.32 (7.63)
**Gender**	
Male	103 (37.2)
Female	168 (60.6)
Other	6 (2.2)
**Marital status**	
Married	107 (38.6)
Partnered	81 (29.2)
Single	89 (32.1)
**Highest educational level**	
Middle School	1 (0.4)
High School	100 (36.1)
Bachelor’s degree	105 (37.9)
Graduate/Master	28 (10.1)
MD/PhD	38 (13.7)
Other ^2^	5 (1.8)
**Vaccine acceptance**	
Provax	197 (71.1)
Antivax	80 (28.9)

^1^ Mean and Standard Deviations. ^2^ No response.

## Data Availability

The data presented in this study are available on request from the corresponding author.
